# Bayesian Geostatistical Modeling of Malaria Indicator Survey Data in Angola

**DOI:** 10.1371/journal.pone.0009322

**Published:** 2010-03-23

**Authors:** Laura Gosoniu, Andre Mia Veta, Penelope Vounatsou

**Affiliations:** 1 Swiss Tropical Institute, Basel, Switzerland; 2 Consultória de Serviços, Estudos e Pesquisas - COSEP Consultória, LDA, Luanda, Angola; New York University School of Medicine, United States of America

## Abstract

The 2006–2007 Angola Malaria Indicator Survey (AMIS) is the first nationally representative household survey in the country assessing coverage of the key malaria control interventions and measuring malaria-related burden among children under 5 years of age. In this paper, the Angolan MIS data were analyzed to produce the first smooth map of parasitaemia prevalence based on contemporary nationwide empirical data in the country. Bayesian geostatistical models were fitted to assess the effect of interventions after adjusting for environmental, climatic and socio-economic factors. Non-linear relationships between parasitaemia risk and environmental predictors were modeled by categorizing the covariates and by employing two non-parametric approaches, the B-splines and the P-splines. The results of the model validation showed that the categorical model was able to better capture the relationship between parasitaemia prevalence and the environmental factors. Model fit and prediction were handled within a Bayesian framework using Markov chain Monte Carlo (MCMC) simulations. Combining estimates of parasitaemia prevalence with the number of children under 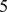 we obtained estimates of the number of infected children in the country. The population-adjusted prevalence ranges from 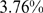 in Namibe province to 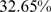 in Malanje province. The odds of parasitaemia in children living in a household with at least 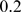 ITNs per person was by 41% lower (CI: 14%, 60%) than in those with fewer ITNs. The estimates of the number of parasitaemic children produced in this paper are important for planning and implementing malaria control interventions and for monitoring the impact of prevention and control activities.

## Introduction

Malaria is a major public health problem and the principal cause of morbidity and mortality in Angola. In 2004 there were reported 3.2 million cases of malaria, two-thirds of which occurred in children under 5 years of age ([1]). It is estimated that malaria accounts for 35% of the overall mortality and 60% of hospital admissions of children under five years ([2]), yet by 2003 only 2% of under-5s used insecticide-treated nets ([3]). Angola recently emerged from almost three decades of civil war (1985–2002) which interrupted malaria control activities and severely damaged the health infrastructure. Only 30% of the population currently has access to government health facilities ([4]). The available statistics for the burden of malaria are not reliable because of the poor case reporting system and the lack of nationally representative malaria surveys. Accurate maps of the distribution of malaria together with human population data are valuable tools for generating valid estimates of the infected population.

Because of the long period of civil unrest, no recent nationwide, population-based surveys have been carried out in Angola. Therefore there are no accurate estimates of the burden of malaria in the country or nationwide coverage and use of key malaria control measures. A Multiple Indicator Cluster Survey (MICS) conducted by UNICEF in 2000 estimated household ownership of insecticide treated nets (ITN) at less than 10% ([5]). Indoor residual spraying (IRS) activities have been very limited in the country over the last 10 years ([6]).

The 2006–2007 Angola Malaria Indicator Survey (AMIS) is the first nationally representative household survey in the country assessing coverage of the key malaria interventions and measuring malaria-related burden among children under 5 years of age. The survey was conducted with the support of National Malaria Control Program (NMCP) within the Ministry of Health (MOH) and was implemented by the Consultória de Serviços e Pesquisas – COSEP, Consultória, Lda. and the Consultória de Gestão e Administração em Saúde–Consaúde, Lda organizations. The focus of the survey was to assess the prevalence of malaria and anemia among children under 5 and estimate the use of ITNs and intermittent preventive treatment (IPT) for malaria among pregnant women, as well as coverage of IRS.

In this paper, the Angola MIS data were analyzed to produce the first smooth map of parasitaemia prevalence based on contemporary nationwide empirical data in the country. Non-linear relationships between parasitaemia risk and environmental predictors were modeled by categorizing the covariates as well as by employing two non-parametric approaches, the B-splines and the P-splines. To identify the method which best models the non-linear environmental effects, three model validation approaches were applied. To account for geographical correlation in the malaria data, Bayesian geostatistical models with household-specific random effects were fitted. These models introduce spatial correlation into the covariance matrix of the random effects. Model fit was performed within a Bayesian framework using Markov chain Monte Carlo (MCMC) methods, providing accurate estimates of the model parameters and their standard errors.

## Materials and Methods

### Study area

Angola is located along the south Atlantic in southwest Africa and covers 1,246,620 km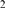. It is bordered by the Republic of Congo on the north, the Democratic Republic of the Congo on the north-east, Zambia on the east and Namibia on the south. The ethnic population of Angola is composed of Ovimbundu (37%), Mbundu (25%), Bakongo (13%), mestiços - mixed European and native African - (2%), European (1%) and other ethnic groups (22%). Malaria is a major public health problem in Angola. *Plasmodium falciparum* is the parasite responsible for more than 90% of malaria infections. The transmission is greatest during the rainy season and peaks between January and May. The prevailing malaria vectors are *Anopheles gambiae*, *Anopheles funestus* and *Anopheles melas*. National Malaria Control Programme in Angola assisted by several international programs (such as Global Fund to Fight AIDS, Tuberculosis and Malaria (GFATM), the President's Malaria Initiative (PMI), UNICEF etc.) adopted several key strategies for malaria control including increasing the coverage of antimalarial treatment, possession and use of insecticide-treated nets (ITNs) and use of intermittent preventive treatment (IPT) among pregnant women to at least 60%.

### MIS Data

#### Ethic statement

The survey protocol was submitted to and approved by the Ethical Review Committee at the National Malaria Control Program and the Institutional Review Board (IRB) of Macro International. Written informed consent was obtained from the respondents participating in the survey.

**Figure 1:**
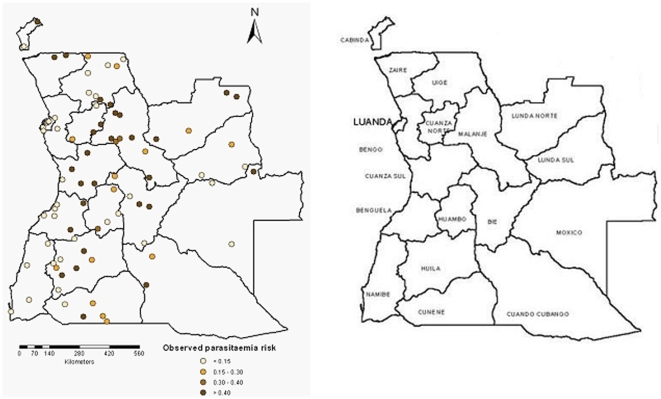
Observed parasitaemia prevalence in children less than 5 years old from the MIS carried out in Angola at 92 locations (left). Angola province map (right).

The 2006–2007 AMIS sample was selected in three stages stratified by epidemiological regions (hyperendemic: in the north and in the lowlands of the Atlantic coast, mesoendemic stable: in central and eastern areas and mesoendemic unstable: in southern and eastern areas) and urban/ rural status, with sampling probability proportional to the population size of selected communes. Further details on sample design are available in [7]. Fieldwork was conducted between November 2006 and April 2007, during the rainy season. The survey collected information from 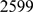 households. A total of 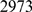 women of reproductive age (15–49 years) were interviewed on various health issues like reproduction, pregnancy, intermittent preventive treatment of malaria and treatment of fever in children. Information on the demographic characteristics of the population as well as on households facilities (water source, toilet facilities and flooring materials) and assets (radio, bicycle, bed nets) were included in the household questionnaires. As part of the AMIS, blood samples from all children age 6–59 months were collected and tested for anemia and malaria. In addition, fieldworkers collected information on the use of IRS and mosquito bets to prevent malaria. To determine the spatial coordinates of the surveyed communes three different databases were used: Geographic Names Information System (GNIS) (http://geonames.usgs.gov), GEOnet Names (http://earth-info.nga.mil/gns/html) and Google Earth (Google, Seattle, USA). The MIS Angola communes included in the survey are shown in Figure 1.

### Environmental and climatic data

**Figure 2:**
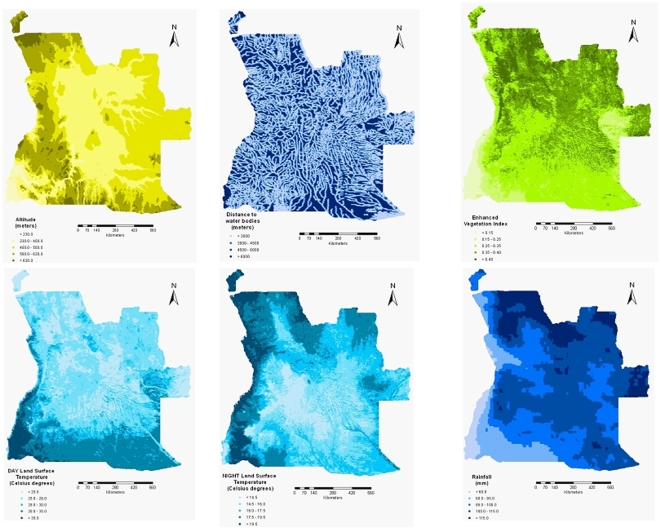
The distribution of environmental factors in Angola.

Environmental and climatic data were extracted from satellite images. Vegetation and land surface temperature (LST) data were obtained from Moderate Resolution Imaging Spectroradiometer (MODIS) at 1km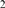 spatial resolution for the period November 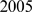
–April 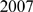. Dekadal rainfall data were available at 8km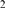 resolution via Africa Data Dissemination Service (ADDS). Although the temporal and spatial resolution of the rainfall data may seem high, these are reasonable resolutions because usually precipitations cover rather large areas and the interest lies in the variation of rainfalls between locations and not the absolute value. Permanent rivers and lakes were extracted from Health Mapper and the shortest Euclidean distance between the centroid of each commune and the closest water body was calculated in ArcGIS version 9.1 (ESRI; Redlands, CA, USA). Altitude data were obtained from an interpolated digital elevation model (DEM) from the U.S. Geological Survey - Earth Resources Observation and Science (USGS EROS) Data Center at a spatial resolution of 1km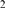. The geographical distributions of the environmental factors are displayed in Figure 2.

### Bayesian geostatistical modeling

We assumed that the malaria status 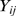 of child 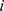 at location 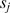, which takes a value of 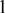 if the child has malaria and 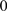 otherwise, follows a Bernoulli distribution 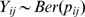. Logistic regression models were run in Stata/SE version 10 (Stata Corporation, College Station, Texas) to identify the environmental and climatic factors significantly associated with malaria risk. The exploratory analysis revealed a non-linear relationship between covariates and malaria prevalence. To account for this non-linearity, three methods were employed. First, the risk factors were categorized, using cut offs based on scatter plots. Two further non-parametric approaches based on spline smoothing were applied, B-splines and P-splines. The advantage of the last two methods is that, instead of specifying the function that describes the change in the response variable when one or more explanatory variables are changed, they estimate this function from the data. The probability of having malaria 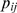 is given by 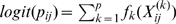, where 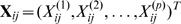 is the vector of 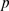 associated environmental predictors observed at location 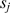 and 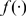 is a smooth function of the covariates.

Bayesian geostatistical models with location-specific random effects were fitted to estimate the degree of spatial correlation in the malaria risk data and to assess the effect of different covariates in the presence of geographical heterogeneity. Spatial dependence was modeled by assuming that the random effects 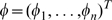 are distributed according to a multivariate normal with mean 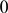 and the covariance between two locations 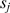 and 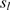 an exponential parametric function of the distance 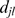 between them, 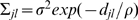. The 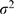 is the spatial variation and 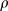 is the parameter which controls the rate of correlation decay with increasing distance. An additional set of location-specific random effects 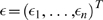 were included to account for unexplained non-spatial variation. They are assumed to be independent, arising from a normal distribution 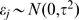, where 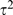 is called the nugget effect and accounts for the non-spatial variation in the malaria risk data. Due to the large number of model parameters, Markov chain Monte Carlo ([8]) simulation methods were used for model fitting.

Model validation was employed to select among the three Bayesian models capturing non-linearity in the relationship between parasitaemia prevalence and the environmental covariates. For the purpose of validation, the model fitting is usually carried out on 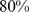 of the data locations and the comparison of the predictive ability of the models on the remaining 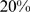 of locations. In our case, the models were fitted to a randomly selected subset of 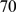 locations (training set). The remaining 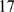 locations (test points) were used for validation. The predictive performance of the three models was assessed by calculating: i) a Bayesian “p-value” analogue, ii) the Kullback-Leibler difference between observed and predicted malaria risk and iii) probability of including the true risk in its predicted highest posterior density interval (HPDI) in relation to the width of this interval. The first two approaches are described in detail in [9]. In particular, for each test location the area of the predictive posterior distribution which is more extreme than the observed data was calculated and the model with the “p-value” close to 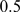 is considered the one with the best predictive ability. The Kullback-Leibler distance between the observed prevalence and the predictive posterior distribution is calculated by 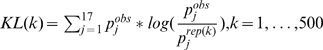, where 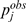 is the observed risk at test location 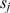 and 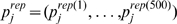 are 500 replicated data points from the predictive distribution at test site 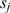. The model with the smallest Kulback-Leibler value is considered the best. For the third validation approach, 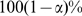 HPDIs were obtained for different confidence levels 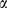, with 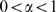. The percentages of test locations with observed malaria risk falling inside these intervals were computed, as well as the width of the HPDIs. The best model is considered the one with the highest coverage of the test locations and the narrowest HPDIs.

To assess the effects of malaria interventions in the country after adjusting for environmental factors, the model with the best predictive ability was fitted again including additional covariates, that is the socio-economic status, the indoor residual spraying status of each household and the number of ITNs per person in each household. The best model in terms of its predictive ability was further employed to predict malaria prevalence at unsampled locations using Bayesian kriging ([10]). The predictions were based only on the relationship between the malaria risk and the environmental factors since data on the socio-economic status and the malaria interventions are not available at high resolution for the whole country. Predictions were made for 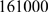 pixels covering on a regular grid the whole area of Angola. In addition, the estimates of the number of children 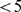 years old with malaria parasites were obtained by multiplying for each pixel the number of children with the parasitaemia risk. The number of children 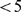 years old in Angola were acquired from the International Data Base of the U.S. Census Bureau, Population Division for the year 2006. The spatial analysis was implemented using software written by the authors in Fortran 95 (Compaq Visual Fortran Professional 6.6.0) using standard numerical libraries (NAG, The Numerical Algorithms Group Ltd.).

## Results

**Figure 3:**
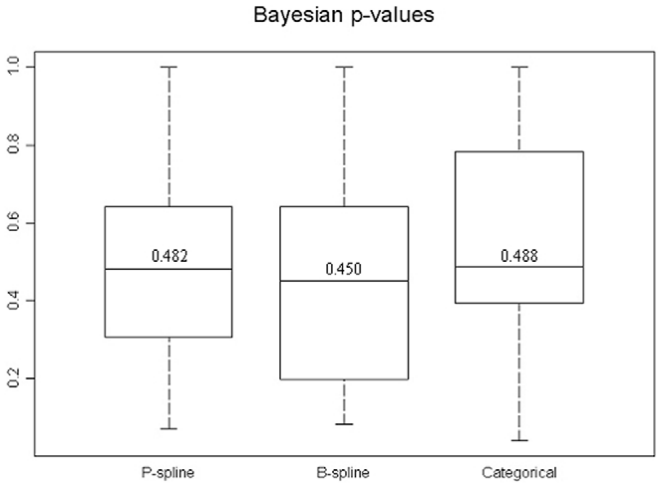
The distribution of Bayesian p-values for the three Bayesian geostatistical approaches that model the non-linear environmental effect. The box plots display the minimum, the 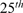, 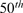, 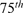 and the maximum of the distribution.

**Figure 4:**
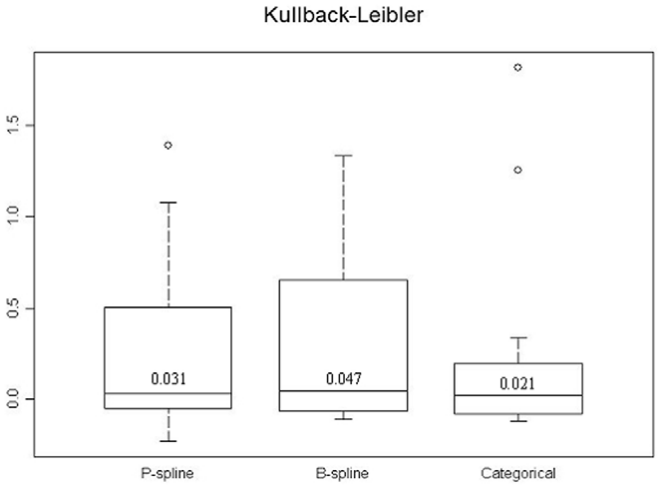
The distribution of the Kullback-Leibler measure for the three Bayesian geostatistical approaches that model the non-linear environmental effect.

**Figure 5:**
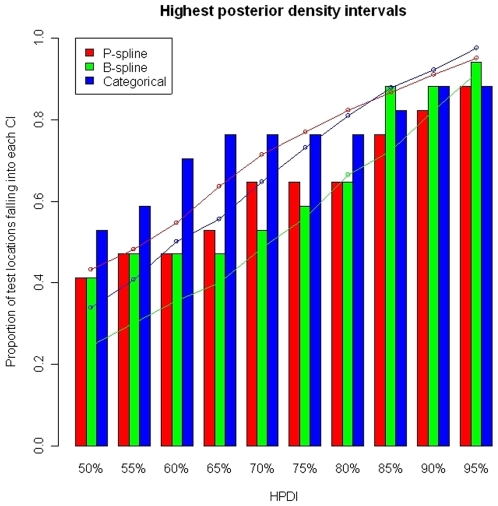
Percentage of test locations with malaria prevalence falling in the predicted highest posterior density intervals (bar plots). The wideness of the predicted highest density regions (line plots). Non-linear environmental effects are modeled via P-splines (red), B-splines (green) and categorizing the covariates (blue).

The results of the model validation are presented first since both inference and predictions are based on the model with the best predictive ability. Figure 3 shows the distribution of the “p-values” of test locations estimated by the P-spline model (left box plot), B-spline model (center box plot) and the model with categorized covariates (right box plot). The median “p-value” of the former model is closer to 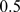, suggesting that this is the best model. However, there is no significant difference between the median “p-values” of the categorical and B-spline models. The distribution of the Kulback-Leibler difference measure is shown in Figure 4. The categorical model has the smallest Kulback-Leibler value. The accuracy of the prediction as well as the width of the HPDI's are shown in Figure 5. In terms of coverage, the best performance is found for the categorical model. However, the B-spline model shows narrower HPDI's compared with the categorical model. This might explain its failing performance to include the true parasitaemia risk in its HPDI. Based on the results of the model validation, the categorical model was employed for estimating the relationship between malaria prevalence and environmental/climatic factors and produce a smooth map of parasitaemia prevalence.

**Figure 6:**
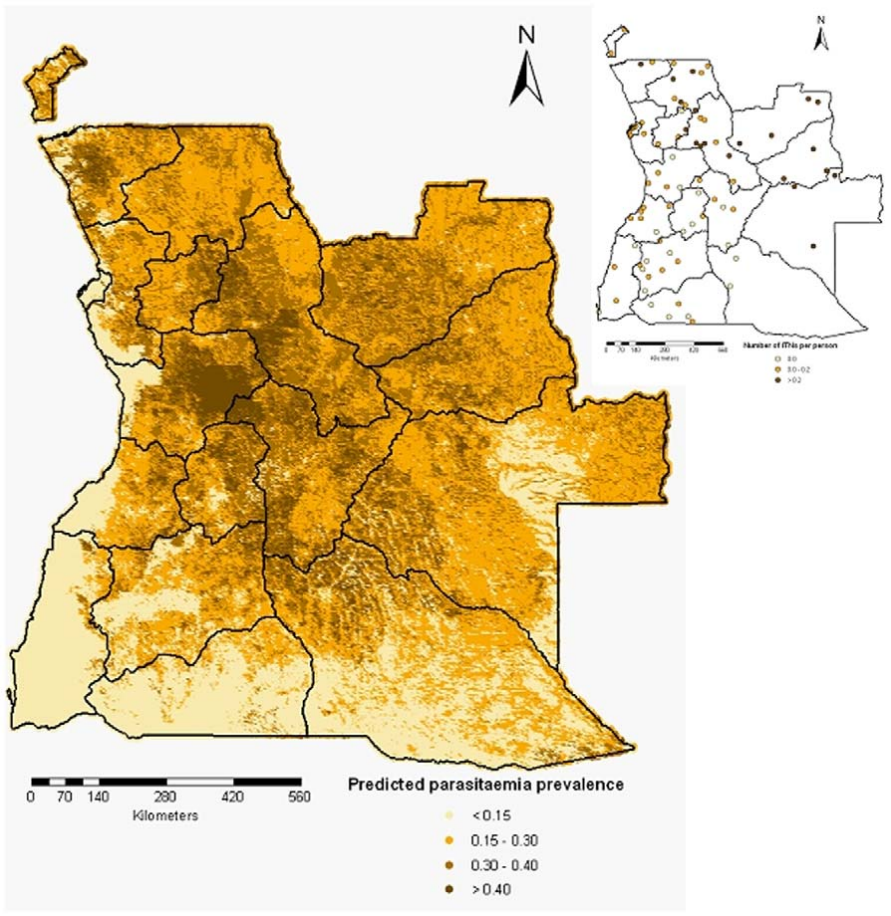
Smooth map of the parasitaemia risk in children 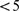 years in Angola.

**Figure 7:**
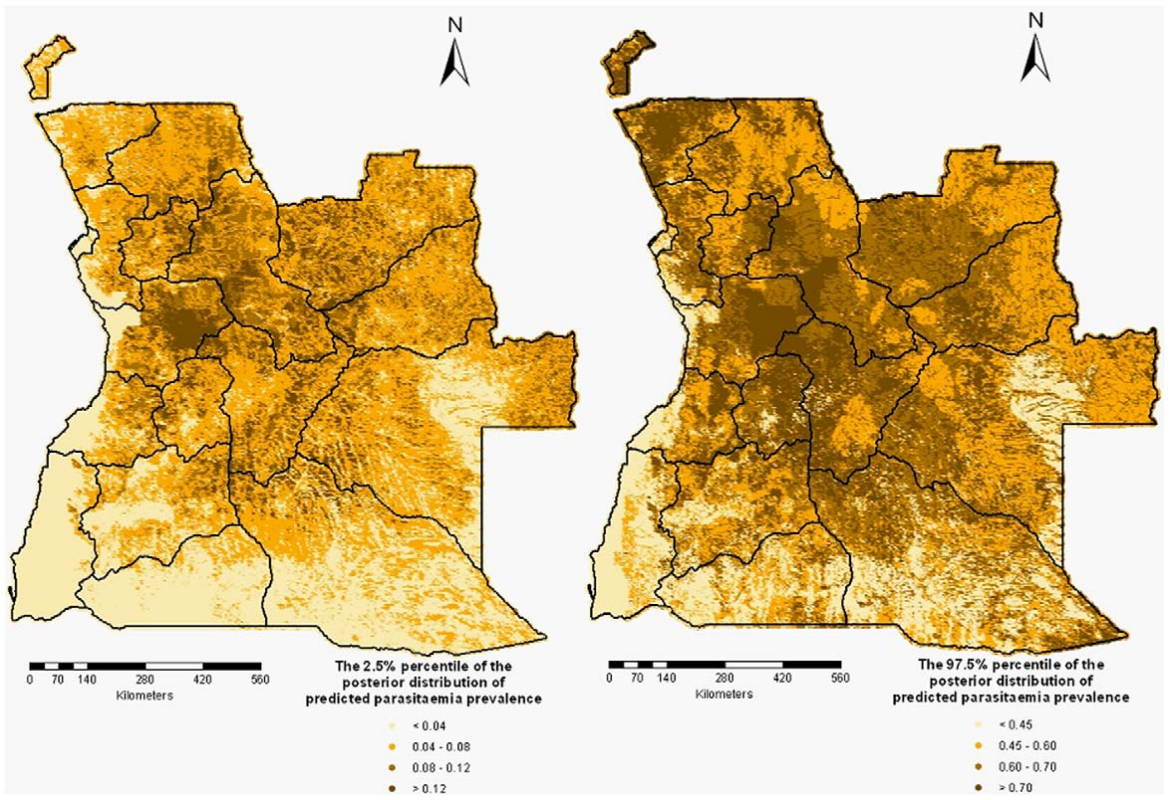
The lower (left) and upper (right) percentiles of the posterior distribution for the predicted malaria parasitaemia.

The predicted parasitaemia risk obtained by employing the Bayesian kriging over a grid of around 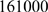 pixels is shown in Figure 6. The estimates are based on the Bayesian geostatistical categorical model with environmental and climatic predictors and correspond to the median of the posterior predictive distribution. The predicted parasitaemia risk varies between 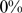 and 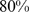, while the observed risks range from 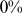 to 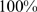, with only 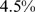 of the data having a risk larger than 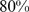. Low levels of parasitaemia prevalence (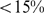) are observed in the Namibe province, the areas along the Atlantic Ocean, the central part of Moxico province and parts of Huila, Cunene and Cuando Cubango provinces. Relatively high prevalences were predicted for Zaire, Malanje, Cuanza Sul, Bie and Muambo provinces. The lower (2.5%) and upper (97.5%) percentiles of the posterior distribution corresponding to the predicted malaria risk are depicted in Figure 7.

Table 1 shows the effects of environmental and climatic factors, socio-economic status and malaria control interventions on the parasitaemia risk in children less than 5 years old. The estimates are obtained from the bivariate model and the Bayesian geostatistical models with the predictors environmental factors, socio-economic status and the malaria interventions. The bivariate analysis shows that all covariates are significantly associated with the risk of parasitaemia. In particular, temperature, altitude, distance to the nearest water body, socio-economic index and indoor residual spraying were negatively associated with the parasitaemia prevalence. A positive association was observed between Normalized Differenced Vegetation Index (NDVI), rainfall and number of ITNs per person. The results of the Bayesian geostatistical models including only the environmental factors show that none of the covariates remained significant. When the effect of socio-economic status and malaria control interventions was added, only the fourth quintile of the socio-economic index (less poor) was negatively associated with the disease prevalence, as well as the number of ITNs per person (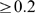).

**Table 1:** Association of parasitaemia risk with environmental/climatic factors, socio-economic status and malaria interventions, resulting from the bivariate and multivariate non-spatial models and the Bayesian geostatistical model.

Variable	Bivariate non-spatial model		Geostatistical model		Geostatistical model including SES and interventions	
	OR	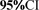	OR	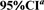	OR	95% CI^a^
*Day LST (* 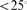 *C)*	1.0		1.0		1.0	
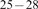	0.67	(0.53,0.84)	0.93	(0.29,3.04)	0.85	(0.30,2.44)
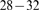	0.44	(0.30,0.65)	0.95	(0.20,4.47)	0.66	(0.15,2.91)
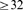	0.15	(0.09,0.25)	0.57	(0.08,3.82)	0.57	(0.12,2.80)
*Night LST (* 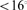 *C)*	1.0		1.0		1.0	
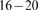	1.10	(0.85,1.42)	0.61	(0.18,1.96)	0.64	(0.20,1.92)
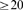	0.34	(0.25,0.47)	0.73	(0.15,3.37)	1.04	(0.24,4.21)
*NDVI (* 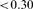 *)*	1.0		1.0		1.0	
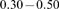	2.29	(1.25,4.17)	0.80	(0.10,5.87)	0.69	(0.11,4.21)
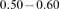	8.66	(5.02,14.94)	2.88	(0.30,24.36)	2.74	(0.35,22.58)
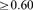	10.62	(6.28,17.94)	3.92	(0.40,33.94)	4.34	(0.53,36.74)
*Rainfall (* 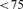 *mm)*	1.0		1.0		1.0	
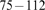	8.16	(4.53,14.70)	4.61	(0.63,34.67)	2.69	(0.49,15.44)
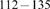	10.97	(6.11,19.71)	3.34	(0.43,26.87)	2.81	(0.43,18.57)
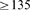	10.80	(6.00,19.43)	3.41	(0.44,27.54)	2.26	(0.37,14.83)
*Altitude (* 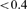 *km)*	1.0		1.0		1.0	
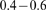	0.62	(0.49,0.79)	0.64	(0.22,1.81)	0.58	(0.22,1.47)
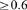	0.96	(0.71,1.30)	0.96	(0.22,4.14)	1.35	(0.42,4.49)
*Distance to nearest water body (* 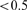 *km)*	1.0		1.0		1.0	
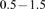	0.74	(0.50,1.10)	0.93	(0.17,5.27)	0.92	(0.18,4.81)
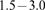	0.30	(0.20,0.44)	0.50	(0.10,2.56)	0.61	(0.13,2.86)
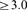	0.36	(0.26,0.49)	0.72	(0.17,2.96)	0.67	(0.17,2.66)
*Socio-economic status*						
Most poor	1.0				1.0	
Very poor	0.77	(0.60,0.99)			0.96	(0.66,1.39)
Poor	0.31	(0.23,0.43)			0.66	(0.36,1.20)
Less poor	0.10	(0.06,0.17)			0.38	(0.16,0.85)
Least poor	0.16	(0.09,0.28)			0.77	(0.31,1.84)
*IRS*	0.22	(0.10,0.47)			0.49	(0.16,1.37)
*ITNs per person (* 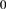 *)*	1.0				1.0	
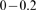	1.28	(0.91,1.80)			0.79	(0.48,1.28)
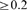	1.26	(1.0,1.58)			0.59	(0.40,0.86)

*^a^*Credible intervals.

The posterior estimates of the spatial parameters are given in Table 2. The decay parameter 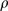 had a posterior median of 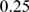 (95% CI: 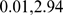) which, in the current exponential setting, corresponds to a minimum distance for which the spatial correlation becomes negligible of 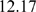 km (95% CI: 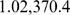). This indicates a quite strong spatial correlation in the parasitaemia data. The estimate of the spatial variance parameter (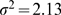, 95% CI: 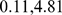) is higher than the estimate of the non-spatial variance (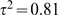, 95% CI: 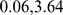).

**Table 2:** Posterior estimates of spatial parameters.

Spatial parameter	Median	95% CI^a^
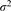	2.13	(0.11, 4.81)
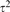	0.81	(0.06, 3.64)
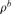	0.25	(0.01, 2.94)

*^a^*Credible intervals.

*^b^*Based on the decay parameter 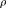, the range parameter 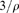 (in km) is calculated.

**Figure 8:**
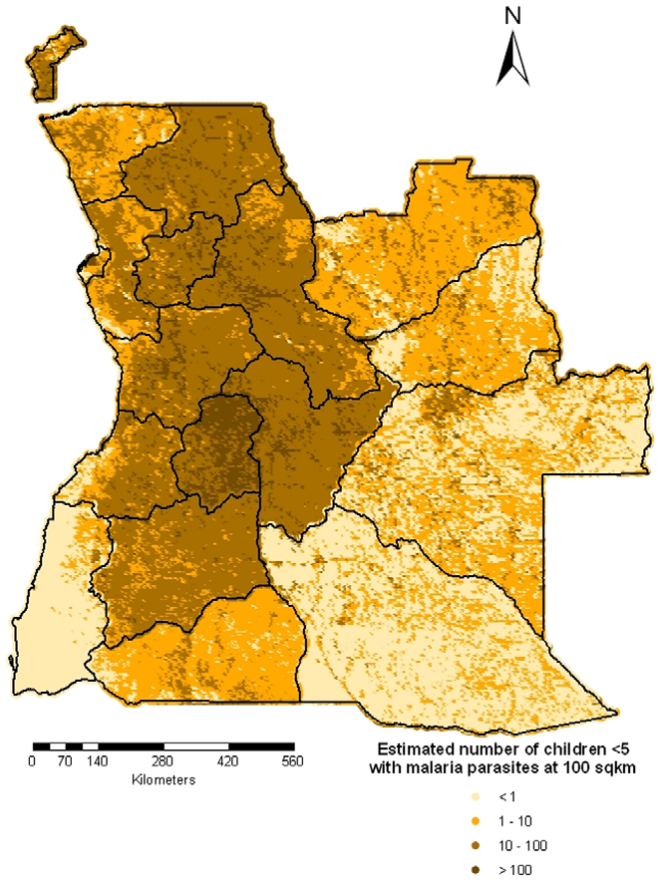
Estimated number of children less than 5 years old with malaria parasites in Angola.

The predicted number of children 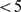 years old with malaria parasites is shown in Figure 8. The estimates of number of children 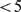 with malaria parasites at the provincial level are presented in Table 3. We observe that after adjusting for population distribution, the mean risk of parasitaemia in the country dropped from 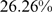 to 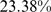. Namibe province, which is the second lowest urban populated area has the lowest population-adjusted prevalence (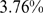). The province with the highest population-adjusted risk is Malanje (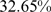), one of the most populated provinces in the country.

**Table 3:** Estimates of the number of children less than 5 years old with malaria parasites at province level.

Province	Number of children <5	Infected children	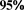 CI	Model-based prevalence	Model-based prevalence adjusted for population
Bengo	33703	8124	(1815,21119)	23.40%	24.10%
Benguela	160008	24604	(516,125302)	21.49%	15.38%
Bie	197908	62979	(2197,184612)	33.43%	31.82%
Cabinda	19926	5119	(105,18333)	26.90%	25.69%
Cuando Cubango	25856	4685	(123,22676)	18.95%	18.12%
Cuanza Norte	82365	25414	(893,77611)	30.63%	30.86%
Cuanza Sul	116591	35817	(1401,107114)	32.50%	30.72%
Cunene	48451	5433	(151,41044)	12.52%	11.21%
Huambo	246028	74375	(2785,228185)	31.54%	30.23%
Huila	144474	27610	(808,124421)	22.10%	19.11%
Luanda	284414	25015	(149,158791)	11.53%	8.80%
Luanda Norte	50845	13553	(419,46721)	30.43%	26.66%
Luanda Sul	22690	6557	(178,20710)	29.25%	28.90%
Malanje	176638	57670	(2335,163306)	32.62%	32.65%
Moxico	57179	13817	(286,49300)	24.64%	24.16%
Namibe	32026	1204	(24,19547)	7.79%	3.76%
Uige	155405	43108	(1208,143887)	27.85%	27.74%
Zaire	62031	12978	(315,53128)	29.65%	20.92%
TOTAL	1916540	448063	(106469,1156997)	26.26%	23.38%

## Discussion

In this paper we have analyzed the parasitaemia data from the first nationally representative malaria survey in Angola to identify significant predictors associated with the parasitaemia risk and to produce a contemporary smooth map of the disease risk in the country. The 2006/2007 Angola MIS included information on malaria-related burden among children under 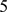 years of age, malaria control intervention (ITNs and IRS) coverage and background characteristics (i.e. household assets). One of the goals of the project is to provide baseline data against which to measure the effectiveness of on-going control interventions. Using data on the population distribution in the country, estimates of the number of children 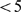 years old infected with malaria parasites were obtained. These could be compared with estimates from future MIS surveys in Angola to evaluate the progress of intervention programmes.

The map of parasitaemia risk in Angola presented in this paper is the first map based on contemporary nationwide empirical data in the country. [11] produced a climatic suitability map of malaria transmission in sub-Sahara Africa, but this was based only on biological constraints of temperature and rainfall on malaria parasite and vector development. Recently a number of prevalence maps based on historical malaria survey data in Mali ([12],[9]) and West Africa ([13],[14]) have been produced. However, these maps do not reflect the current malaria situation at a specific location, which could be influenced by control measures or human activities on which historical information are not available. In addition, the historical field survey data are heterogeneous in season and age since they are collected in different seasons and they covered populations with non-standardized and overlapping age groups. These constraints make it difficult to consider seasonality and age adjustment in malaria mapping. MIS are a new source of malaria data which address the drawback of the historical data. They are contemporary nationally representative data, collected all in the same season (usually the season with highest malaria transmission), covering standardized age groups. In addition, the MIS collect information on malaria intervention coverage and wealth status of the population at household level, allowing adjustment for these factors. The first country which successfully completed the MIS is Zambia. The data were analyzed by Riedel et al. (unpublished) and a first parasitaemia map of Zambia was produced.

An important aspect that needs to be highlighted regarding the MIS in general and AMIS in particular is that they are all conducted during the transmission season, therefore the parasitaemia risk maps resulting from the analysis are specific to this time period and may not reflect the situation of malaria for the whole year. Since all future MIS are planned to be carried out also during the highest transmission season, these maps are providing the baseline situation of the disease against which the forthcoming maps could be compared to assess the effectiveness of intervention programs.

Angola has three main agro-ecological zones. The northern region is characterized by a humid tropical climate, with an annual rainfall over 2000 mm. Our model predicted one of the highest levels of parasitaemia risk in this region (Zaire province). The central region has a temperate tropical climate. This area is characterized by an annual rainfall ranging from 1250 mm to 1500 mm. Estimates of parasite prevalence reach the highest level in this region (provinces of Malanje, Cuanza Sul and Bie). The south and south-west part of the country, where our model predicted the lowest disease risk, is characterized by a dry climate ranging from tropical desert to tropical dry, with low annual rainfall (20 mm average). Based on this comparison, we can conclude that the parasite prevalence map produced in this paper is in line with the distribution of agro-ecological zones in Angola.

Malaria is endemic all over Angola, however the country is stratified into three regions based on levels of endemicity ([7]). Malaria is hyperendemic in the north, mesoendemic stable in central and eastern areas, and mesoendemic unstable in southern and eastern areas. The geostatistical model predicted low disease risk in the south and south-east part of the country which is classified as mesoendemic unstable. Low risk is also observed in the central-west coastal and south-west regions which are classified as mesoendemic stable. This may be explained by the highest temperature and low rainfall and vegetation. There is a noticeable low risk area in the central-east possible due to low level of vegetation. The highest risk was observed in the central and north part of the country which is classified as hyperendemic and mesoendemic unstable regions. The estimated prevalence map does not indicate clearly the geographical limits between the hyperendemic and the mesoendemic stable zones. Also the ITN coverage (presented in upper right hand side of Figure 6) reflects the endemicity level of malaria. Few ITNs per person are present in the unstable areas, while the number of ITN per capita increases as we move towards the northern part of the country which has perennial malaria transmission.

After adjusting for the population distribution, we observe that the country is split in two main regions: the eastern part with low population-adjusted prevalence and the western part with the highest level of disease risk, with the exception of Namibe province in the south-west. Based on the population-adjusted risk map, control intervention should be concentrated mainly in the central-south, central and north part of the country. Special attention should be given to the Malanje province which has the highest estimated number of infected children.

Malaria data are geographically correlated due to common exposures, therefore the spatial correlation must be taken into account. Most MIS are carried out on rather small number of locations (around 100); however the locations cover the whole country. Scarcity of the data in certain areas would introduce large prediction errors. The advantage of the modelling method we have used in this paper is that it provides estimates of the prediction uncertainty which are shown in Figure 7. The modeling approach employed in this study was based on the assumption of stationarity, that is the spatial correlation was considered a function of only the distance between locations and was independent of the locations themselves. The results obtained from the Bayesian geostatistical models were similar to the findings of Riedel et al. (unpublished). In particular, the authors found no relationship between environmental/climatic factors and the parasite prevalence in Zambia. In addition, the only intervention measure significantly associated with a decrease in parasitaemia risk was bednet ownership, while IRS had no significant effect on the disease risk. Our analysis indicates also no relationship between IRS within the last 24 months and the parasitaemia risk. More than 0.2 ITNs per person in a household was found to be significantly associated with a decreased risk of parasitaemia. The similar results regarding the effects of malaria intervention measures between Angola and Zambia were to be expected considering the similar coverage of ITNs among children younger than 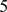 years in the two countries ([15]). Unfortunately, at the moment there is no database on the distribution of malaria intervention coverage at high spatial resolution, therefore these factors may not be used when predicting malaria risk at unsurveyed locations. Including these covariates in the kriging would significantly increase the predictive ability of the models.

The estimates of the number of parasitaemic children produced in this paper are very important for planning and implementing control interventions and for monitoring the impact of prevention and control activities. Information on the number of infected children could be compared to existing levels of service provision to identify under served populations and to target interventions to high priority areas.
